# Corrigendum to “Herpesvirus BACs: Past, Present, and Future”

**DOI:** 10.1155/2019/6870815

**Published:** 2019-07-08

**Authors:** Charles Warden, Qiyi Tang, Hua Zhu

**Affiliations:** ^1^Department of Molecular Medicine, City of Hope National Medical Center and Beckman Research Institute, Duarte, CA 91010, USA; ^2^Department of Microbiology/AIDS Research Program, Ponce School of Medicine, 395 Zona Industrial, Reparada 2, Ponce, PR 00716-2348, USA; ^3^Department of Microbiology and Molecular Genetics, UMDNJ-New Jersey Medical School, 225 Warren Street, Newark, NJ 07101-1709, USA

In the article titled “Herpesvirus BACs: Past, Present, and Future” [1], there was an error in the second paragraph in the “Studies of Specific Herpesvirus BACs” section, where the text reading “For example, AD169, Towne, and TB40 are considered the “clinical” BAC strains whereas Toledo, FIX, PH, and TR are considered to be the “laboratory” BAC strains [28]” should be corrected to “For example, AD169, Towne, and TB40 are considered the “laboratory” BAC strains whereas Toledo, FIX, PH, and TR are considered to be the “clinical” BAC strains [28].”
